# Predicting Organometallic Intermediates in the Surface-Assisted Ullmann Coupling of Chrysene Isomers

**DOI:** 10.3390/molecules29071553

**Published:** 2024-03-30

**Authors:** Jakub Lisiecki, Paweł Szabelski

**Affiliations:** Department of Theoretical Chemistry, Institute of Chemical Sciences, Faculty of Chemistry, Maria Curie-Skłodowska University in Lublin, Pl. M.C. Skłodowskiej 3, 20-031 Lublin, Poland; jakublisiecki96@gmail.com

**Keywords:** chrysene isomers, adsorption, Ullmann coupling, functional molecules, Monte Carlo simulations, polymerization

## Abstract

On-surface polymerization of functional organic molecules has been recently recognized as a promising route to persistent low-dimensional structures with tailorable properties. In this contribution, using the coarse-grained Monte Carlo simulation method, we study the initial stage of the Ullmann coupling of doubly halogenated chrysene isomers adsorbed on a catalytically active (111) crystalline surface. To that end, we focus on the formation of labile metal-organic precursor structures preceding the covalent bonding of chrysene monomers. Four monomeric chrysene units with differently distributed halogen substituents were probed in the simulations, and the resulting precursor structures were compared and quantified. Moreover, the effect of (pro)chirality of chrysene tectons on the structure formation was elucidated by running separate simulations in enantiopure and racemic systems. The calculations showed that suitable manipulation of the halogen substitution pattern allows for the creation of diverse precursor architectures, ranging from straight and winded chains to cyclic oligomers with enantiopure, racemic, and nonracemic composition. The obtained findings can be helpful in developing synthetic strategies for covalent polymers with predefined architecture and functionality.

## 1. Introduction

Polymer synthesis in confining environments has recently been found to be a promising route to low-dimensional covalent structures with tailorable properties. In this field, diverse on-surface heterogeneous catalytic reactions, including Ullmann coupling, have been most frequently used to achieve control over the structural features of the final polymeric product [1,2,3]. One advantage of this approach has been the increased possibility of steering self-organization and subsequent bonding of monomers by suitable tuning of molecular size, shape, and functionalization pattern. In this case, the reduced dimensionality of available reaction space (adsorbed overlayer) makes the bond formation facilitated and more effective due to lowered conformational freedom of adsorbed reactants and also to substantial reduction of their translational and rotational modes. In consequence, precise designing of low-dimensional polymeric constructs can often be based on the intrinsic properties of a monomer at play. This strategy has been demonstrated to be highly effective for numerous adsorbed systems, in which covalently bonded structures, such as graphene nanoribbons [4,5], polyaromatic nanowires [6,7], oligomers (also cyclic) [8,9], and many others [1,8,10,11], have been created using Ullmann coupling and several remaining heterogeneous catalytic reactions (e.g., Glasser or Suzuki coupling [12,13]). 

In an on-surface Ullmann reaction, aryl halides are adsorbed on a catalytically active metallic substrate (usually Cu, Ag, and Au) and, upon either spontaneous or thermally activated scission of halogen atoms, transformed into reactive species capable of forming covalent bonds. In some instances, the covalent bonding is preceded by the organometallic intermediate sustained by labile interactions between surface adatoms and aromatic radicals [14,15,16]. The occurrence of this superstructure is often beneficial, as various local defects can then be healed by breaking and reformation of relatively weak organometallic connections. In consequence, adsorbed assemblies with improved quality (size, extent of ordering, connectivity, etc.) can be obtained and transformed into persistent polymeric products—usually by heating the system so that metal-organic bonds are converted into covalent ones. Because in this process the structure of the intermediate is often directly transmitted to the polymer, it is thus desirable to understand and control the formation of the corresponding organometallic precursor right from the start. This task is especially important as after heating, the covalent linkage of the monomers eliminates the possibility of any further changes to the polymer.

To create new polymeric structures via the Ullmann coupling reaction, a plethora of organic monomers have been used to date, including terphenyl linkers and polyaromatic hydrocarbon (PAH) units with differently attached halogen atoms [1,2,3]. As in practice, the number of halogenated isomers of a given building block can be large, preliminary selection of the optimal tecton, able to form a polymer with predefined properties, may therefore be tedious. This task is even more difficult for the monomers which are able to adopt multiple adsorbed conformations—resulting, for example, in mirror-image forms (surface enantiomers) which can occur under 2D confinement [11]. In consequence, choosing the best candidate out of a large pool of isomers requires several test syntheses, subsequent polymerization reactions, and Scanning Tunneling Microscopy (STM) imaging of the formed products.

To facilitate the design of low-dimensional polymeric constructs formed on surfaces, computer-aided methods have recently been used. This refers especially to quantum chemical calculations focusing on bonding mechanisms and reaction paths of polymer formation [15,17] and to more self-assembly-oriented methods, such as the Monte Carlo [18,19,20,21,22] and molecular dynamics [23] simulations. While quantum methods (e.g., density functional theory, DFT) correspond to zero-Kelvin temperature and are usually limited to a few adsorbed molecules (interacting pairs or clusters), MC calculations allow for modeling of large molecular assemblies comprising thousands of units at finite temperatures. The latter method makes it possible to trace the structural evolution of the adsorbed phase under variable conditions and identify the main macroscopic products of the self-assembly. In consequence, MC simulations can be effectively used to determine the structure–property relation in diverse molecular systems, owing to the very simple ways of modification of the intrinsic properties of the building blocks and interactions between them. One of the main advantages here is the easy exploration of how molecular features, such as size, shape, and intramolecular distribution of active centers (e.g., halogen substituents), in a given monomer affect the architecture of the corresponding precursor and subsequent polymeric product. 

In the previous works, we developed a coarse-grained MC model of the formation of metal-organic precursors in the surface-assisted Ullman coupling of PAH isomers [24,25,26]. Those studies focused on the relation between monomer properties (shape, size, halogen distribution) and morphology of the resulting metal-organic precursors. This simplified approach enabled the analysis of a huge number of probe tectons and resulted in precursor structures (thus, potential covalent polymers) with diverse topologies, such as isolated clusters, cyclic oligomers, straight and winded chains, ladders, periodic and aperiodic networks, and even fractal aggregates resembling the Sierpiński triangle [27]. 

In this study, inspired by the recent experimental report on the Ullmann coupling of dihalogenated chrysene monomers [28,29,30,31], we investigate how our model can predict the structure of metal-organic precursors created by molecules of this type. Chrysene is an intrinsically prochiral molecule that provides the opportunity to study chirality aspects at the nanoscale and fabricate chirality-selective structures, such as graphene nanoribbons. Due to the high potential for application, previous experimental studies have focused on analyzing the metal-organic and covalent structures that chrysene can form on the surface of copper, gold, and silver [28,29,30,31]. To that purpose, Scanning Tunneling Microscopy (STM) has been used, and the formation of both homochiral (copper) and heterochiral (silver and gold) metal-organic precursor chains has been observed. The extended chain structures realized experimentally with the particular isomer 6_12-dibromochrysene (DBCh) prompted us to ask whether similar adsorbed architectures can be obtained using different positional isomers of DBCh. Moreover, the unique intrinsic prochirality of DBCh has provided the opportunity to observe how the molecular (chiral) properties are transmitted to the resulting metal-organic superstructures. To validate our theoretical approach, we first performed calculations for the 6_12-DBCh isomer and compared the simulated results with their experimental counterparts. Next, the modeling was extended to a few selected chrysene isomers bearing two halogen substituents in qualitatively different relative positions. Our main objective here was to provide insights related to the monomer synthesis (positions of halogens in chrysene) and the direction of the self-assembly towards precursors with predefined structural properties.

## 2. Results and Discussion

Before we proceed with the simulated results, let us notice that the chrysene (**ch**) molecule is intrinsically prochiral, that is, it can be adsorbed in the two mirror-image conformations, here denoted arbitrarily by *R* and *S*. Importantly, this property refers to any (mono-, di-, etc.) halogenated isomer of **ch**, and for that reason, two qualitatively different situations related to the composition of the adsorbed phase can be considered. Specifically, for the chrysene tectons, both enantiopure and mixed overlayers are possible to model, with a special focus on the experimentally relevant racemic composition. From a practical point of view, because the adsorption of *R* and *S* is not biased, the natural composition of the adsorbed phase should be racemic, as it has been confirmed for many other chiral systems on surfaces. On the other hand, the creation of the enantiopure adsorbed structures requires forcing a molecule of **ch** to adopt one conformation (say *S*). Even though this task would be much more difficult for real systems (e.g., introduction of extra substituents to **ch**) herein we study and compare the enantiopure and racemic self-assembly of di-X-chrysene isomers. In this case, our main goal is to determine how the presence of the opposite enantiomer influences the formation of metal-organic precursors comprising the prochiral **ch** tectons. Realization of the above objective will also highlight possible differences between the subsequent covalent enantiopure polymers and their racemic counterparts. Accordingly, in the following for each isomer, we discuss the enantiopure overlayers set side by side with the corresponding racemic systems. Specifically, we examine the self-assembly of four dihalogenated chrysene isomers with distinct substitution patterns, that is 6,12 (**ch6_12**); 3,6 (**ch36**); 2,6 (**ch26**); and 6,11 (**ch6_11**). 

### 2.1. Validation of the Model: 6,12-Dibromochrysene

**Figure 1:**
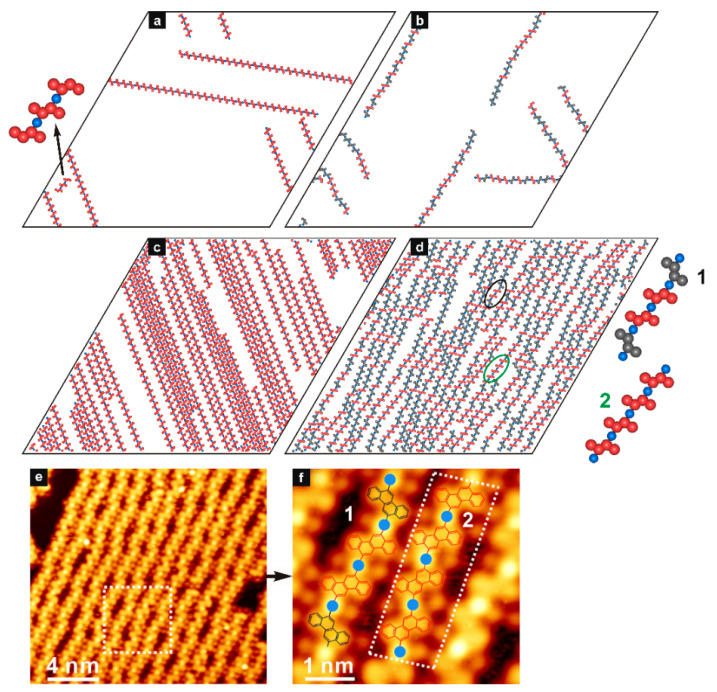
Adsorbed structures simulated for the enantiopure (**a**,**c**) and racemic (**b**,**d**) systems comprising 100*S* (**a**), 700*S* (**c**), 50*R* + 50*S* (**b**), and 350*R* + 350*S* (**d**) molecules of **ch6_12** mixed with 100 and 700 metal atoms, respectively. The magnified chain fragments illustrate exemplary sequences of *R* and *S* observed in the simulations, as highlighted in panel (**d**); T=0.01, θ=0.0125 (**a**,**b**) and θ=0.0875 (**c**,**d**). Parts (**e**,**f**) are the experimental STM images of the racemic metal-organic precursor formed by the molecules 6,12-dibromochrysene (DBCh) adsorbed on Ag(111) at 423 K. Reproduced and adapted from ref. [28]. The monomer sequences 1 and 2 marked in the zoomed fragment (**f**) are identical to their theoretical counterparts presented next to panel (**d**).

We start the discussion with the **ch6_12** isomer which corresponds to 6_12-dibromochrysene (DBCh)—a molecule whose Ullmann coupling on metallic substrates has been recently studied experimentally [28,29,30,31]. This previously tested molecule [32] was included here primarily to validate our model using the available experimental data. Figure 1 presents the results of the enantiopure self-assembly of **ch6_12**(*S*) simulated for 100 tectons and 100 metal atoms (a) and 700 tectons and 700 metal atoms (c). 

As seen in part (a), the adsorbed units created straight chains, in each of which the contributing enantiomers had the same orientation. This uniform alignment of the contributing enantiomers *S* is shown in the inset to panel (a), and it agrees with the local structure postulated for the homochiral chains created by 6,12-dibromochrysene (DBCh) on the Ag(111) surface [28]. Because of the low adsorbate density (θ=0.0125), the growing chains were able to find enough room to propagate along the three main directions—imposed by the symmetry of the lattice. When the adsorbed phase became more crowded (θ=0.0875), the homochiral chains of **ch6_12**(*S*) preferred to run in the same direction, as demonstrated in panel (c) of Figure 1. The dense parallel packing of chains allowed for the maximization of their length and eliminated termination caused by chain crossing. This effect was more and more pronounced with the gradual increase in *θ*, as seen in the snapshots from Figure S1 of the Supporting Information section. Interestingly, the creation of structurally the same homochiral chains has been observed experimentally for the adsorption of DBCh on the Cu(111) surface [28]. In this case, however, the overlayer comprised both surface conformers *R* and *S* (due to unbiased adsorption), which spontaneously segregated into the corresponding homochiral chains. The resolution of the enantiomers of DBCh has been attributed to the subtle geometric effects resulting from the slight incompatibility between the copper lattice and the alignment of the adatoms cementing mixed metal-organic chains. This substrate-induced effect has not been detected for the other surfaces including Au(111) [29] and Ag(111) [28].

Let us now focus on the results obtained for the racemate of **ch6_12**, which are presented in the right part of Figure 1. The snapshots shown therein correspond to the low (b) and high (d) surface coverages used previously for the enantiopure self-assembly (i.e., here 50*R* + 50*S* and 350*R* + 350*S* molecules, respectively). The adsorbed overlayer simulated for the lower density comprised sparse *R* + *S* mixed chains having a weakly wavy shape. The observed deviation from linearity resulted from the atactic character of the chains, in which the units *S* and *R* were distributed entirely randomly. The formation of homochiral sequences (*S* or *R*) in these chains produced relatively short straight fragments, while further incorporation of another molecule of the opposite type disturbed the unidirectional growth. In consequence, some limited bending of the mixed chain occurred, and in fact, this effect was strongly reduced because of the collinear interaction directions assigned to **ch6_12** (see Figure 1). Similar observations refer to the densely packed systems presented in Figure 1d. Like for the self-assembly of pure *S* shown in Figure 1c, at the high adsorbate density, the mixed *S*/*R* chains tend to align in parallel to achieve the largest possible length, thus minimizing the number of uncoordinated monomers. As seen in the racemic system, unidirectional packing is less effective in comparison to pure *S*, resulting from the bigger effective width of the mixed chains.

**Figure 2:**
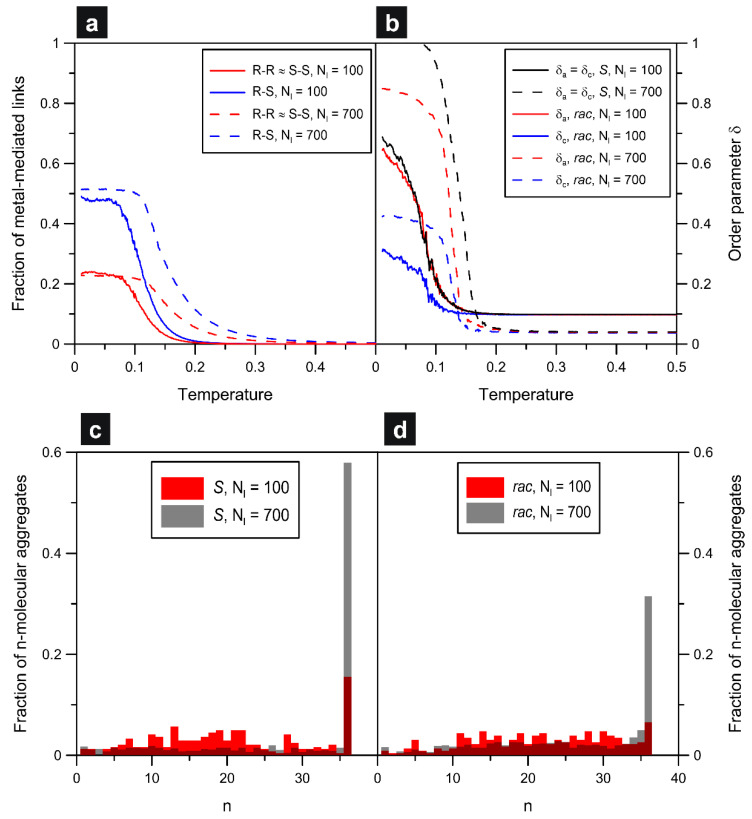
Effect of temperature on the fraction of homo- (*R*-*R*) and heterochiral (*R*-*S*) links (**a**) and on the arm and core orientational order parameters (*δ_a_*, *δ_c_*) (**b**) calculated for the adsorbed overlayers comprising 100 (blue, θ=0.0125) and 700 (red, θ=0.0875) molecules of **ch6_12**. Dashed and solid lines correspond to the enantiopure (*S*) and racemic systems, respectively. Panels (**c**,**d**) present the associated size distribution functions characterizing aggregates built of *n* molecules, obtained at T=0.01, for the enantiopure and racemic systems, respectively.

To demonstrate the correct predictive abilities of our model, in Figure 1e,f, we show the corresponding STM images obtained for the polymerization of 6,12-dibromochrysene (DBCh) on the Ag(111) crystalline surface [28]. As it follows from the comparison of the modeled racemic structures (Figure 1d) and their experimental counterparts (Figure 1e,f), the simulated configurations agree closely with the STM data. In particular, the diverse random sequences of *R* and *S* observed for DBCh occurred also in the simulations, highlighting the lack of energetic preference for the formation of homochiral (*R*-metal-*R* and *S*-metal-*S*) vs. heterochiral (*R*-metal-*S*) links on Ag(111). This can be easily noticed from the magnified chain fragments 1 and 2 shown in the STM snapshots and from the corresponding theoretical structures. To quantify and prove the purely random distribution of enantiomers *S* and *R* along the modeled chains, in Figure 2, we plotted fractions of metal atoms coordinated homo- (*R*-*R*, *S*-*S*) and heterochirally (*R*-*S*) as functions of temperature. As seen in part (a) of the figure, the homochiral and heterochiral fractions increased when the temperature went to zero, and they finally reached about 0.22 (red) and 0.50 (blue), respectively. 

The obtained values are very close to the statistics characterizing entirely random hypothetical mixing of the enantiomers (assuming unbiased homo/heterochiral linkage), that is, 0.25 (*R*-*R* and *S*-*S*) and 0.5 (*R*-*S*), respectively. The aforementioned limiting tendencies (T→0) are independent of the adsorbate density, and they demonstrate the same random way of arranging monomers *S* and *R* in chains formed at low and high *θ*. From Figure 2a, for the racemate with $N_{l}=700$ (dashed lines), it can be also noticed that the onset of growth of the homo- and heterochiral fractions is clearly shifted towards higher temperatures. The explanation for this effect lies in the increased chance for the formation and stabilization of metal-mediated connections in the more crowded overlayer where the linkage can be quite effective even at elevated temperatures, as compared to $N_{l}=100$. 

Panel (b) of Figure 2 presents additional quantitative characteristics which reflect the way of ordering in the modeled systems. The parameters *δ_a_* and *δ_c_* plotted therein as functions of temperature are the order parameters which measure the degree of co-directional alignment of molecular arms (*a*) and cores (*c*) of **ch** (see definitions in the Supplementary Materials). Briefly, these descriptors are close to 1 when the corresponding molecular parts (arms or cores) are all parallel to each other, and *δ_a_* and *δ_c_* tend to zero when the allowed molecular orientations become equally probable. For the enantiopure **ch6_12**(*S*) systems, *δ_a_* = *δ_c_* (here arm orientation univocally determines core orientation), and the drop in temperature causes the sudden growth of the population of uniformly oriented enantiomers, as demonstrated by the black curves. This effect reflects the formation of homochiral chains, which for the denser system ($N_{l}=700$) run all in parallel, so that *δ_a_* approaches 1 when *T* goes to zero (dashed line). The limiting value of this parameter, calculated for 100 molecules of **ch6_12**(*S*), is noticeably smaller (around 0.7) and results directly from the presence of the differently oriented shorter chains visible in Figure 2a. In this case, even though the enantiomers have the same orientation within one chain, the diverse chain directions reduce *δ_a_* effectively. A similar situation refers to the racemic self-assembly, for which the contact values of *δ_a_* predicted for 100 and 700 molecules are noticeably different, that is, 0.65 and 0.85, respectively.

However, these estimates are lower compared to their enantiopure counterparts, especially for the denser overlayer (0.7 and 1.0). Here, the racemic chains, due to some (limited) bending ability are able to avoid crossing with orthogonal chains and persist even in the more crowded adsorbed phase. This effect can be clearly seen in the additional snapshots from Figure S1.

Contrary to the enantiopure overlayers, the parameters *δ_a_* and *δ_c_* obtained for the racemates of **ch6_12** are not equal, as demonstrated by the corresponding temperature dependencies plotted in Figure 2b. Specifically, the contact values of *δ_c_* predicted for $N_{l}=100$ and $N_{l}=700$ are about two times smaller than the analogous values of *δ_a_*. This dependence reflects the statistically equally frequent occurrence of the two main core orientations (i.e., *S* and *R*) in the chains, corresponding to random packing of the enantiomers in these structures. Despite the same orientation of the arms of all monomers in a mixed chain, the units *S* and *R* contribute with oppositely oriented cores (mirror-image directions) and thus substantially reduce *δ_c_*. The larger contact value of this parameter predicted for $N_{l}=700$ (blue dashed line) is a direct consequence of the enhanced unidirectional alignment of the chains in the denser adsorbed phase. 

The two bottom panels of Figure 2 present the aggregate size distribution functions calculated for the enantiopure (c) and racemic (d) overlayers of **ch6_12**. As it follows from these plots, in both cases, the increased adsorbate density promotes the formation of longer chains—often running across the entire surface. This effect manifests in the strong increase in the peaks located at n=37—corresponding to the number of molecules in the longest (straight) chain that is possible for the assumed *L*. As mentioned previously, the main reason here is the confining effect, which forces the chains to grow in parallel and avoid crossing-induced termination. Specifically, for the homochiral chains, the sustaining metallic nodes lie on a straight line that is rotated by ±9°, relative to the angularly closest main lattice direction. However, for the racemic composition, the chains are bent with no common growth direction.

### 2.2. 3,6-Di-X-chrysene

**Figure 3:**
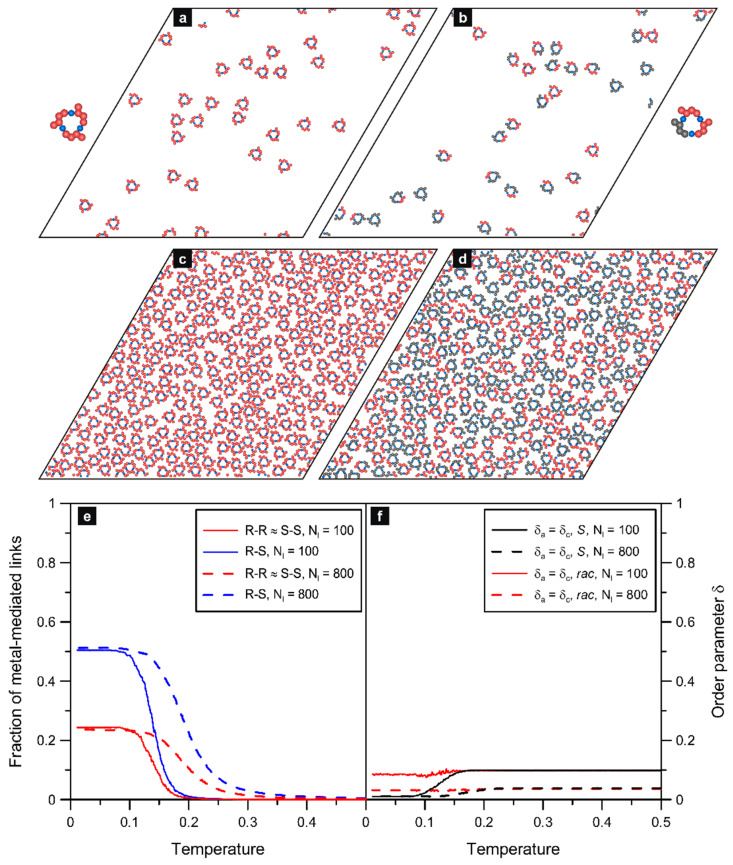
Simulated enantiopure (**a**,**c**) and racemic (**b**,**d**) overlayers comprising 100*S* (**a**), 800*S* (**c**), 50*R* + 50*S* (**b**), and 400*R* + 400*S* (**d**) molecules of **ch36** mixed with 100 and 800 metal atoms, respectively; T=0.01. Typical trimeric structures are shown enlarged next to the corresponding snapshots. The bottom part shows the effect of temperature on the fraction of homo- (*R*-*R*) and heterochiral (*R*-*S*) links (**e**) and on the arm and core orientational order parameters (*δ_a_*_,_ *δ_c_*) (**f**) calculated for these systems. The enantiomers *S* and *R* are shown in red and grey, respectively; metal atoms are colored in blue.

In this section, we discuss precursor structures predicted for the three selected isomers, which, to the best of our knowledge, have not been probed experimentally. In this context, the results presented in the following can help create new chrysene-based polymeric structures with controlled properties. We start with the isomer **ch36**, which is revealed to be able to form metalorganic connections with the simplest architecture. Panel (a) of Figure 3 shows the enantiopure overlayer comprising 100 molecules of **ch36**(*S*), which created trimeric ring aggregates. Those triangular oligomers were uniform in size and shape, and the qualitative picture of the adsorbed overlayer did not change upon further increase of the surface coverage, as seen in panel (c) of Figure 3 ($N_{l}=800$, T=0.01). A clearly more complex structure formation was observed when the other enantiomer of **ch36** was introduced to the system. In this case, the racemic self-assembly resulted also in the creation of tri-molecular ring oligomers, however, with varied enantiomeric composition. Part (b) of Figure 3, corresponding to $N_{l}=100$, illustrates the occurrence of homochiral 3*R* and 3*S* aggregates, accompanied by heterochiral units 2*R* + *S* and 2*S* + *R*. As seen in this panel, the mixed-ring structures occurred more frequently compared to the homochiral ones. From simple statistical considerations, it follows that the relative abundance of homo- and heterochiral trimers should be equal to 1:3 (one and three distinguishable configurations for 3*R* and 3*S* and for 2*R* + *S* and 2*S* + *R*, respectively). Indeed, this ratio is approximately reflected in the bar charts presented in Figure S2.

The expected dominating contribution of the heterochiral oligomers of **ch36** was also observed in the case of a dense overlayer comprising 400 *R* + 400 *S* molecules, presented in panel (d) of Figure 3. Moreover, the way the ring trimers were formed is clearly reflected in the corresponding temperature dependencies shown in panel €. The coordination functions plotted therein for the homo- (*R*-*R*) and heterochirally (*R*-*S*) bonded metal atoms confirm the unbiased self-assembly of the trimeric aggregates. Note that at low temperatures, these curves reach about 0.25 and 0.50 ($N_{l}=100$) and 0.21 and 0.53 ($N_{l}=800$), respectively, which agrees with the statistical ratio of 1:2 resulting from the assumed energetic equivalence of metal-mediated links of all types. Specifically, a single aggregate 3*R* or 3*S* contains three metal atoms coordinated homochirally, while in one aggregate 2*R* + *S* or 2*S* + *R*, there are two metal atoms coordinated heterochirally and one metal atom linked with a pair of enantiomers of the same type. Taking into account the aforementioned relative abundance of these aggregates (1:3; *R*-*R*:2*R* + *S*—true also for the mirror-image structures), the average ratio 1:2 of homo- vs. heterochiral metallic nodes in the *rac*-**ch36** overlayer becomes rationalized. The slight deviation from this proportion, in favor of the heterochiral links (0.53), predicted for the dense system with $N_{l}=800$ originates from the smaller effective size of the heterochiral trimers. In this case, at high adsorbate densities, the mixed *R*/*S* aggregates can be packed more effectively in comparison to the homochiral trimers.

**Figure 4:**
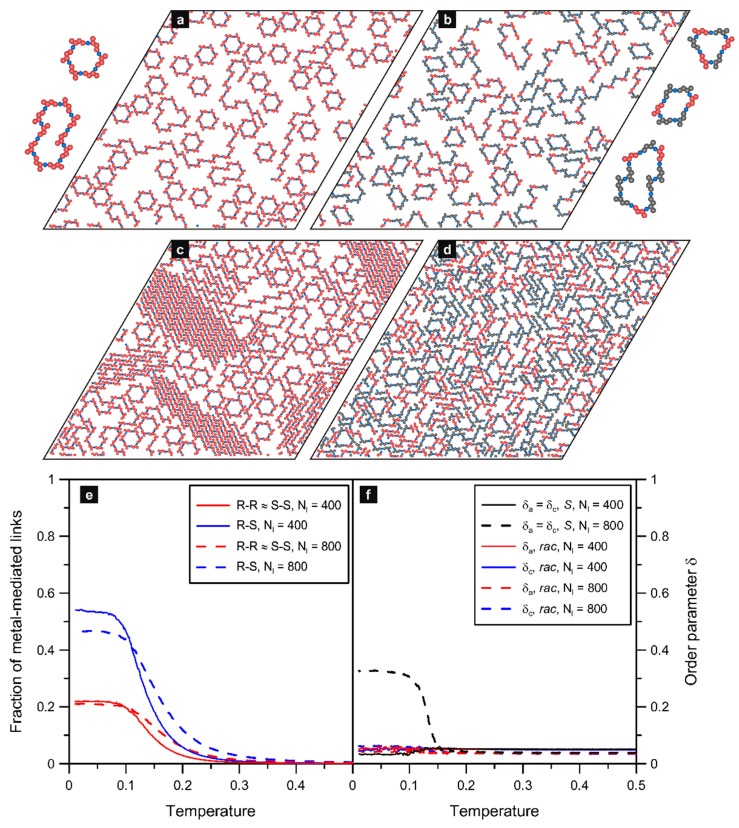
Simulated enantiopure (**a**,**c**) and racemic (**b**,**d**) overlayers comprising 400*S* (**a**), 800*S* (**c**), 200*R* + 200*S* (**b**), and 400*R* + 400*S* (**d**) molecules of **ch26** mixed with 400 and 800 metal atoms, respectively; T=0.01. Typical cyclic structures observed in the simulations are shown next to the corresponding snapshots. The bottom part presents the effect of temperature on the fraction of homo- (*R*-*R*) and heterochiral (*R*-*S*) links (**e**) and on the arm and core orientational order parameters (*δ_a_*, *δ_c_*) (**f**) calculated for these systems. The enantiomers *S* and *R* are shown in red and grey, respectively; metal atoms are colored in blue.

One more accompanying effect which distinguishes the enantiopure self-assembly of **ch36** from its racemic variant is the way in which the corresponding curves *δ_a_* approach T→0. As seen in Figure 4f, this order parameter, calculated for the racemates with $N_{l}=100$ and $N_{l}=800$, remained small and approximately constant as the temperature was gradually decreased. The reason for this residual non-zero value of *δ_a_* is the fluctuations in the number of molecules with different orientations, proportional to $1/\sqrt{N}$. Due to these fluctuations, the orthogonal molecular orientations do not cancel completely, always producing some (small) net effect. At high temperatures (T>0.15), when the aggregates have not yet been formed, the above situation refers to separate molecules. However, below this value of *T*, the enantiomers *S* and *R* create the heterochiral trimers in which they have arms (one *R*-*S* pair) and cores (second *R*-*S* pair) aligned in parallel. This effect introduced some local uncompensation of the arm/core directions so that both *δ_a_* and *δ_c_* should be smaller. From Figure 3f, it can be seen that the contact values of these parameters (i.e., at T→0) were indeed slightly lower compared to the high temperatures (T>0.15). To quantify the extent of this drop, let us use the simple structural argument applied locally to this system. Because in each trimer there are two pairs of molecules with nonparallel arms (and cores), *δ_a_* and *δ_c_* should be equal to 2/3 of the value characterizing the uncorrelated fluctuations of separated molecules (at high temperatures). These theoretical predictions explain qualitatively the relative level of the flat fragments of the curves plotted for T<0.15 and for T>0.15.

In the case of enantiopure self-assembly of **ch36**, the non-zero value of *δ_a_* at high temperatures has the same origin that was discussed earlier for the corresponding racemate. On the other hand, as seen in panel (f) of Figure 3, when *T* drops, contrary to the racemic systems, the curves referring to pure *S* tend toward zero. This observed effect results directly from the internal structure of the trimers, each of which comprises three enantiomers *S* with different (i.e., orthogonal) orientations. As these three equally contributing molecules reset *δ_a_* to zero locally (see Equation (S1) of the Supporting Information section), any repetition of this closed structural motif (also rotated by 60 degrees) does not increase the value of *δ_a_*. The same refers to *δ_c_* because molecular cores in any ring trimer of **ch36** are aligned in three different directions—highly correlated with the arm orientations. In this case, *δ_a_* and *δ_c_* are identical.

### 2.3. 2,6-Di-X-chrysene

The results presented in Figure 4 refer to the next isomer **ch26** in which the assigned interaction directions form an angle of 120 degrees. This special property allowed the molecules of **ch26** to create larger and more complex structures compared to the trimers simulated for **ch36** (c.f. Figure 3). The above effect was observed at low surface coverages ($N_{l}=100$, see Figure S3), and it was even more pronounced at higher values of *θ*. As seen in part (a) of the figure, at the moderate adsorbate density ($N_{l}=400$), the self-assembly of *S* produced mostly six-membered ring oligomers and less abundant loop structures comprising 10 and 15 molecules (two and three joined hexamers, respectively). This result agrees with the structure formation predicted theoretically [24] and observed experimentally [8,9,32] for diverse polyaromatic tectons with analogous 120-degree angular distribution of active centers. For molecules of this type, the self-assembly at moderate surface coverages gives loop oligomers (hexamers, etc.), which can grow effectively because there is still sufficient space available as well as energetically favorable ring closure (eliminating undercoordinated components). However, when the number of adsorbed enantiomers *S* is further increased, the hexameric units can hardly form in the crowded adsorbed phase and, instead, the densely packed chains prevail, as demonstrated in Figure 4c for $N_{l}=800$. Structural coexistence of this kind has been observed, for example, in the case of dibrominated terphenyl molecules adsorbed on the Cu(111) surface [33].

Regarding the racemic self-assembly of **ch26**, part (b) of Figure 4 presents the results simulated for $N_{l}=400$, while part (d) shows analogous images obtained for the adsorbed overlayer with $N_{l}=800$. As it follows from these data, the formation of mixed *R*/*S* precursors is characterized by considerable structural disorder—induced by the increased number of possible bimolecular configurations. In consequence, constructs such as mixed, strongly bent, chains of different lengths, racemic hexamers with a triangular shape, parallelogram nonracemic 2*S* + 4*R* and 2*S* + 4*R* loop aggregates, and sparse homochiral six-membered rings occur in the system. Examples of these typical units are shown in the inset to Figure 4b. The cyclic R/S hexamers, despite quite a sizable excluded area, can be also found in the denser system with $N_{l}=800$ due to increased stability (no unsaturated ends). However, in this case, the dominant structures are the densely packed winded chains, which meander between the cyclic aggregates.

For *rac*-**ch26**, the formation of mixed (racemic and nonracemic) hexamers, combined with the growth of chains having random *R/S* sequences, resulted in a significant increase in the number of heterochiral (*R*-*S*) bonds. Specifically, regarding the cyclic hexamers, each racemic triangular aggregate contributed with six heterochiral bonds, and each nonracemic 2:4 aggregate contributed with four heterochiral and two homochiral links (see the insets for Figure 4b). This effect can be seen in part (e) of Figure 4, in which we plotted the fractions of homo- and heterochiral metal-mediated connections as functions of temperature. The corresponding contact values of the first function (*R*-*R*, T→0) were equal to about 0.21 (red lines) regardless of the surface coverage. However, in the case of the heterochiral links, the plateau of the curve plotted for the denser system ($N_{l}=800$) is shifted down, to about 0.47, relative to the curve predicted for $N_{l}=400$ (with a plateau at about 0.55). This difference can be explained by taking into account the increased number of short chains occurring in the racemate with $N_{l}=800$. In consequence, in this dense overlayer, there are more singly coordinated metallic centers (not contributing to the statistics) terminating the chains, as compared to $N_{l}=400$. Nevertheless, the obtained contact values are close to those characterizing entirely random efficient mixing of the enantiomers, that is 0.25 (*R*-*R*) and 0.5 (*R*-*S*), respectively.

Panel (f) of Figure 4 shows the effect of temperature on the orientational order parameters *δ_a_* and *δ_c_* calculated for the discussed overlayers comprising **ch26**. As seen therein for the enantiopure (*S*) systems with $N_{l}=400$, the corresponding curve has a shape that is qualitatively similar to **ch36**(*S*). Like for that isomer, the self-assembly of **ch26**(*S*) induced a noticeable drop in *δ_a_* when the temperature approached zero. The origin of this effect was already explained for **ch36**, and here it is associated directly with the full local compensation of molecular orientations in a single homochiral hexamer of **ch26**(*S* and *R*). However, unlike **ch36**, the overlayer of **ch26**(*S*) contains additional structures (mainly irregular chains and bigger looped aggregates); therefore, the net compensation effect, and thus the drop in *δ_a_*, was smaller compared to **ch36**. A markedly different situation was encountered in the case of the denser phase ($N_{l}=800$) comprising the closely packed homochiral chains shown in Figure 4c. The strongly directional alignment of enantiomers **ch26**(*S*) in these chains resulted in the significant growth of *δ_a_* at low temperatures (*T* ~ 0.15), up to about 0.35. The aforementioned effects—which appeared clearly for the enantiopure overlayers of **ch26**—are distinctly different from the slight changes in the orientational parameters characterizing the corresponding racemic systems (red and blue lines). In this case, the considerable structural heterogeneity of the adsorbed phase, at both moderate and high density, resulted in the effective randomness of molecular orientations. On the one hand, the presence of sparse homochiral hexamers reduced *δ_a_* and *δ_c_*, but on the other hand, this effect was compensated by the occurrence of (short) homochiral chain fragments *S* and *R*, where these units were aligned in parallel. In consequence, at low temperatures, molecular orientations of the adsorbed enantiomers (due to the random meandering of chains and possible rotation of the loop structures) were subjected to similar fluctuations as in the case of high temperatures, producing only a marginal increase in *δ_a_* and *δ_c_*.

**Figure 5:**
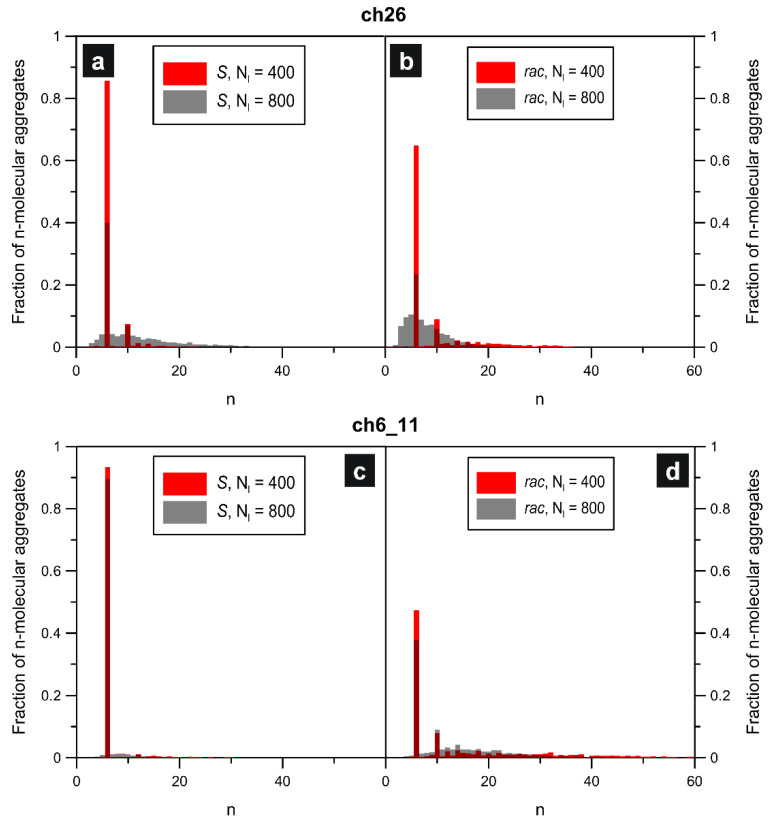
Size distribution functions characterizing aggregates comprising *n* molecules of **ch26** and **ch6_11** obtained at T=0.01 for the corresponding enantiopure (**a**,**c**) and racemic (**b**,**d**) systems, respectively.

To characterize further structural properties of the adsorbed systems comprising **ch26**, we calculated the corresponding aggregate size distribution functions shown in Figure 5. These bar charts illustrate the relative abundance of *n*-molecular aggregates in the modeled systems. As seen in part (a), the highest peaks are centered at n=6 and n=10, regardless of the surface coverage. The aforementioned values correspond to the most numerous homochiral hexamers and the larger ten-membered looped aggregates, respectively (see the insets for Figure 4a). For the less dense overlayer with $N_{l}=400$ (red bars), the marginal signals at n>10 are mainly due to the formation of infrequent but longer-winded chains. A clear effect that can be noticed in part (a) is the significant broadening of the distribution (especially right handed) at the increased adsorbate density ($N_{l}=800$, grey). This broadening was associated primarily with the formation of numerous densely packed homochiral chains having various lengths. Different coverage-dependent changes were observed in the case of the racemic systems of **ch26**, for which the distribution functions were plotted in panel (b) of Figure 5. Here, similarly to pure *R*, the main contribution was associated with the presence of hexameric units (n=6): mixed (see Figure 4b) and much less frequent homochiral. For $N_{l}=400$ (red bars), the second most abundant structures were the closed *R* + *S* forms comprising ten enantiomers (n=10), exemplified in Figure 4b. However, for this less dense system, the distribution function extended up to n<40 due to occasional growth of strongly meandering mixed chains of large length. The quite wide distribution calculated for $N_{l}=400$, contrary to pure **ch26**(*S*), became narrower and shifted toward lower *n*s when the number of molecules was increased. The main reason for this opposite result was the inability of the mixed *R*/*S* connections to create long linear-like forms in the dense overlayer. Instead, the adsorbed molecules formed shorter-winded chain fragments whose growth was terminated quickly in the crowded environment. In consequence, additional clearly marked peaks appeared in the distribution for *n*s even smaller than six. 

### 2.4. 6,11-Di-X-chrysene

**Figure 6:**
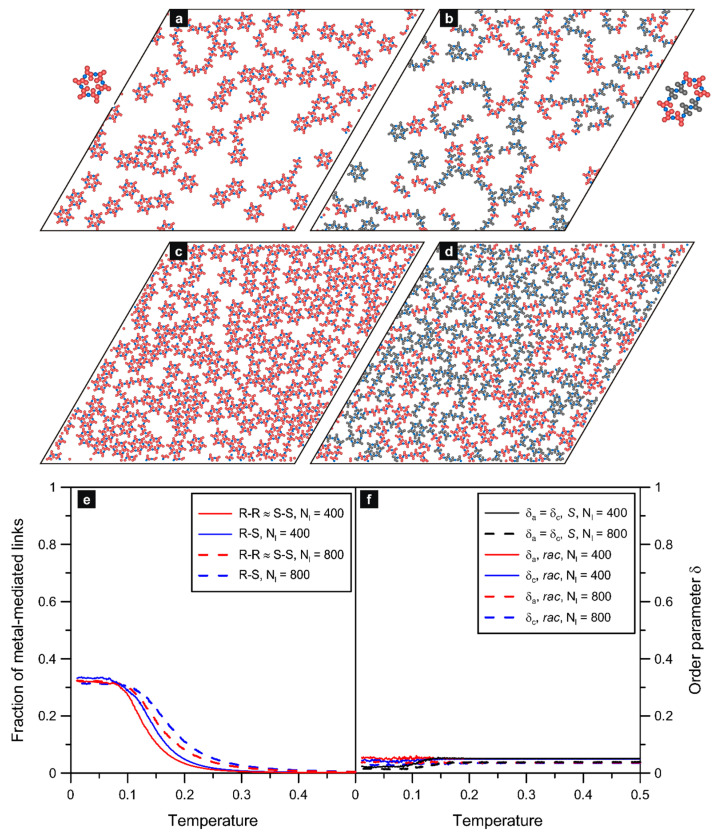
Simulated enantiopure (**a**,**c**) and racemic (**b**,**d**) overlayers comprising 400*S* (**a**) and 800*S* (**c**) and 200*R* + 200*S* (**b**) and 400*R* + 400*S* (**d**) molecules of **ch6_11** mixed with 400 and 800 metal atoms, respectively; T=0.01. The bottom part shows the effect of temperature on the fraction of homo- (*R*-*R*) and heterochiral (*R*-*S*) links (**e**) and on the arm and core orientational order parameters (*δ_a_*_,_ *δ_c_*) (**f**) calculated for these systems. The enantiomers *S* and *R* are shown in red and grey, respectively; metal atoms are colored in blue.

In the last example, we discussed the isomer **ch6_11**, which, like **ch26**, has two interaction directions at an angle of 120 degrees, but contrary to the previous tecton, these interaction directions are both assigned to the molecular core. As will be shown in the following, this difference has important implications, especially for the racemic self-assembly. Let us start with the enantiopure systems: panels (a) and (c) of Figure 6 present snapshots of the enantiopure overlayers comprising 400 and 800 molecules of **ch6_11**(*S*), respectively. As can be seen in part (a), the self-assembly resulted in local structures which were qualitatively similar to their counterparts obtained for **ch26**. Specifically, the most visible ordered constructs are the star-shaped hexamers accompanied by sparse short chains and single loops built of 12 molecules (see the inset). The hexamers comprise molecules whose arms protrude from the central ring (formed by the six metal atoms) having a diameter that is smaller than for **ch26**. This property makes the star aggregates ready for denser packing thanks to the interdigitation of their spiky arms. In consequence, even at the high adsorbate density (c), the molecules of **ch6_11**(*S*) can still create the hexamers, but, contrary to **ch26**, the formation of chains is then strongly suppressed (c.f. Figure 4a). 

The decreased tendency for dense packing of the **ch6_11**(*S*) chains results from the presence of closely spaced side fragments (arms), which hamper side alignment of the long chains. In this context, the specific assignment of the interaction directions in **ch6_11**(*S*), resulting in the formation of protruding side chain fragments, enables denser packing of hexamers, and, at the same time, it reduces chain growth and arrangement of the adsorbed molecules into compact domains. Parts (b) and (d) of Figure 6 show the results simulated for the racemic self-assembly of 400 and 800 molecules of ch6_11, respectively.

When comparing these predictions with the corresponding enantiopure overlayers, it can be easily noticed that the presence of the other enantiomer enhances the formation of winded chains with mixed *S*/*R* composition. This effect occurs at both low and high adsorbate densities, and it originates naturally from the greater diversity of bimolecular connections. In this respect, the self-assembly of *rac*-**ch6_11** is qualitatively similar to *rac*-**ch26** (c.f. Figure 4a,c). However, as we mentioned before, there is a substantial difference in the shape and composition of the loop aggregates created in each of these racemic systems. Specifically, in the case of the latter isomer, the molecules were capable of forming small six-membered racemic aggregates with triangular and parallelogram shapes, as shown in the insets for Figure 4b. However, for the first molecule, this situation is not possible, and the smallest (i.e., hexameric) aggregates occurring in *rac*-**ch6_11** are exclusively homochiral, having the same star-shaped architecture as in the corresponding enantiopure systems. The reason for this effect is the aforementioned special location of active centers in **ch6_11**, which prevents dense packing of *S* and *R* inside the star-shaped hexamers. Under such conditions, the enantiomers tend to form slightly larger loop aggregates, mostly comprising ten units (2*S* and 8*R* and vice versa, see the inset). This limited ability to create closed-chain forms is clearly visible against the background of the results obtained for *rac*-**ch26**, whose molecules often created mixed irregular loops built of over a dozen units. 

The marked tendency for the formation of homochiral star hexamers in the racemic overlayers of **ch6_11** is also reflected in the shape of the coordination curves shown in panel (e) of Figure 6. As seen therein, the fraction of homochiral links (*R*-*R* ≈ *S*-*S*) at low temperatures was considerably higher as compared to the previous systems, where efficient mixing of the enantiomers occurred, that is, ~0.33 vs. 0.25, respectively. This effect occurred equally at the moderate ($N_{l}=400$) and high ($N_{l}=800$) adsorbate densities. A visible consequence of the homochiral self-assembly mode was the decrease in the order parameters *δ_a_* and *δ_c_* when the temperature approached zero, which is illustrated in panel (f) of Figure 6. Similar to the small triangular and hexagonal units observed previously for **ch36** and **ch26**, the hexamers of **ch6_11** comprised six molecules whose orientations canceled out and locally reduced both order parameters to zero. However, similarly to **ch26**, the occurrence of (infrequent but still relevant) chain structures modified this picture, resulting in the non-zero residual value of *δ_a_* and *δ_c_* at low temperatures. For the racemic systems, this influence was even more explicit because the creation of chains with uniformly aligned enantiomers *S* and *R* took place quite often (increasing both parameters)—producing only marginal changes in the level of *δ_a_* and *δ_c_* at low and high *T*.

Regarding the associated aggregate size distribution functions, from part (c) of Figure 5, it is seen that for the enantiopure self-assembly, the dominating structures were the hexameric star-shaped units, n=6, accompanied by sporadically appearing longer chains (n<30 for $N_{l}=400$) and small loops (n=10, see the inset for Figure 6a). At the higher adsorbate density ($N_{l}=800$), the formation of longer homochiral chains of *S* was strongly suppressed, so that the distribution became narrower, extending from n=4 to about 15 with the main peak still at n=6. As shown in part (d) of the figure, the presence of the other enantiomer of **ch6_11** induced significant broadening of the distribution functions, as compared to pure *R*, towards larger values of *n*. In this case, the enantiomers *S* and *R* recreated the compact homochiral hexamers, which correspond to the highest peak at n=6. The second most abundant structures here are the mixed ten-membered units (n=10) shown in the inset for Figure 6b. However, under these conditions, the adsorbed molecules also created numerous long meandering chains which contributed significantly to the observed right-sided widening of the distributions. This is especially visible for the less dense system ($N_{l}=400$, red bars), in which mixed chains, even with n>50, were occasionally formed. Like for the enantiopure overlayers of **ch6_11**, the increase in density of the racemate ($N_{l}=800$, grey bars) resulted in a narrowing of the distribution function—resulting mainly from the limited chances of propagation of long *R*/*S* chains in the dense overlayer. 

The results discussed in Section 2 show that the newly probed chrysene tectons (**ch36**, **ch26**, and **ch6_11**) are able to form cyclic metal-organic structures with various shapes and sizes, including triangular, hexagonal, and larger looped connections. Therefore, the main application potential of our study lies in the on-surface synthesis of macrocyclic compounds, which cannot be obtained using conventional bulk phase protocols. Moreover, our calculations demonstrate how the enantiopure versus racemic composition of the adsorbed phase affects the architecture of the resulting precursors. With this information, one can target the synthesis by controlling the enantiopurity of the adsorbed overlayer, for example, through additional functionalization of the monomers to induce them to adopt only one adsorbed conformation (surface enantiomer).

As we have demonstrated, control over the morphology of the considered systems can be achieved in two ways: first, by manipulating the surface coverage, and second, by choosing the enantiomeric composition of the adsorbed phase. Regarding surface coverage, it is generally easier to control experimentally. This parameter had a significant effect on the structures formed by **ch26**(*R*) (rings vs. chains), while it was revealed to be practically irrelevant for the enantiopure systems comprising **ch6_11** (hexamers) and **ch36** (trimers). In the case of the composition of the adsorbed overlayer, simulations showed that the introduction of the other enantiomer resulted in the formation of mixed R/S structures for all of the modeled tectons. For **ch36**, the mixed trimers had an identical pore interior to their enantiopure counterparts but a differently decorated exterior. This indicates that enantiopurity is not an issue when considering the synthesis of trimeric aggregates with a uniform interior structure. On the other hand, racemic composition can be used to induce the formation of cyclic oligomers with an altered shape (*rac-***ch26**) and bigger size (**ch6_11**). The simulations indicated whether the use of one surface enantiomer or a racemic mixture is beneficial from the standpoint of synthesizing polymeric structures with presumed topologies. In real applications, achieving enantiopurity can be much more challenging because it requires the prochiral isomers of chrysene to adopt a specific adsorbed conformation, necessitating additional chemical modification of these molecules.

Our findings are consistent with previous studies on organometallic intermediates, which have demonstrated that the key factor enabling the directed on-surface synthesis of such structures is the appropriate distribution of active centers (halogen substituents) in a monomer. For example, hexagonal rings created by **ch26** have been observed previously in the case of 4,4″-dibromo-m-terphenyl on a Cu(111) surface [9] as well as with other 120°-bent halogenated polyphenyl tectons used for the surface-assisted synthesis of honeycombene and kekulene macrocycles [34]. This directional-bonding approach has also been applied to the fabrication of more complex hexagonal cyclic structures, including cycloarene [35] and triangulene-based nanostars [36]. In the case of trimolecular aggregates of **ch36**, the 60° bonding pattern has been found to be effective in the Ullmann coupling of 3,12-dibromo-7,8-diaza[5]helicene on an Ag(111) surface, resulting in the production of nitrogen-doped nanographene with [18] annulene pores (triangular trimers) [37].

The good agreement between the results simulated for **ch6_12** and the corresponding STM data [28,29,30,31] confirms that simplified directional interaction Monte Carlo modeling is capable of accurately predicting the architecture of organometallic precursors comprising other tectons. This enables the experimental realization of such superstructures.

## 3. Methods

**Figure 7:**
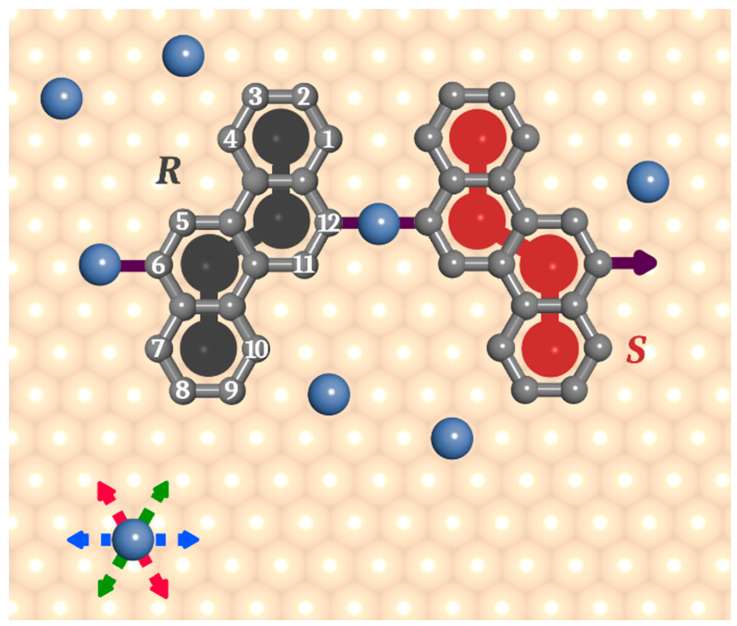
Schematic drawing of the model chrysene molecules used in the simulations. Mirror-image structures (surface enantiomers) of 6,12-dihalogenochrysene (**ch6_12**) are shown as an example, in which the four contributing segments are colored in dark grey (*R*) and red (*S*). The light grey contours show the corresponding benzene rings with locants 1–12 numbered according to the IUPAC convention. The purple arrows indicate directional organometallic interactions corresponding to the position of (scissed) halogen atoms. Metal atoms are represented by blue segments, and they preferentially mediated the linkage of a pair of molecules when the corresponding interaction directions were collinear (illustrated by the pairs of arrows in the same color).

To explore the formation of metal-organic precursors comprising chrysene tectons, we used the coarse-grained MC model developed previously [24,25,26,27]. Specifically, the chrysene molecules, abbreviated here as **ch**, were represented by flat rigid collections of four interconnected segments, each corresponding to one benzene ring, as shown in Figure 1. These monomers were equipped with two differently distributed active interaction centers imitating the halogen substituents (red arrows therein). The molecules were placed on a triangular lattice of equivalent adsorption sites mimicking the catalytically active (111) metallic surface (e.g., gold, silver, copper). For convenience, the lattice constant of the adsorbing surface *a* was assumed to be equal to 1. A single segment of **ch** was allowed to occupy one adsorption site (lattice vertex), and the consecutive segments of **ch** were centered at the next neighbor sites of the lattice (i.e., they were separated by a distance of $a\sqrt{3}$). The active centers of chrysene tectons provided directional interactions with the coadsorbed metal atoms modeled as single segments. The range of these metal–organic interactions was limited to nearest neighbor sites, measuring from the vertices 1–12 of the polyhexagonal contour shaded in Figure 7. The formation of metal–organic links sustaining the precursors was possible only when the interaction directions of the contributing molecules were collinear, as illustrated in the figure. In this case, the energy of interaction assumed for a single link was equal to 2ε, where ε stands for the energy of elementary metal–monomer interaction. Nonlinear 120° linker-metal-linker configurations contributed with energy ε, reflecting the effect of repulsive interactions between active molecular segments located too close to each other. Figure S4 in the Supporting Information section shows the possible one- and bi-molecular metal-linker configurations along with their corresponding energies.

In the calculations, the conventional canonical MC method with Metropolis sampling was used, where the total number of species *N*, temperature *T*, and system size *A* (here area of the adsorbing surface) were fixed [38]. To eliminate edge effects, periodic boundary conditions in both planar directions were imposed. The simulations started with a mixture of *N_l_* molecules of a given isomer and *N_m_* metal atoms distributed randomly on a rhombic fragment of a triangular lattice with side *L* equal to 200*a* (i.e., 200 by 200 adsorption sites); $N=N_{l}+N_{m}$. Next, the adsorbed overlayer was equilibrated in a series of trial moves, each of which corresponded to a single MC step. During one MC step, a molecule or metal atom was picked up at random, and its potential energy in the current position, *U*_o_, was calculated by reckoning the elementary directional interactions (ε) and taking into account the geometric conditions discussed previously (preferred linear →•← links). Next, the selected component was translated to a new random position on the lattice. In the case of chrysene isomers, these units were additionally rotated in-plane by a multiple of 60 degrees. If in the new position, there was enough space (sufficient number of unoccupied adsorption sites), the selected component was inserted therein; otherwise, it was returned to its original site, and the calculation started again. The potential energy of the new configuration, *U*_n_, was determined using the same procedure as for *U*_o_. To accept this configuration, the Metropolis criterion was used with the acceptance probability $p=min[1,\exp\left(-\frac{∆U}{kT}\right)]$, where $∆U=U_{n}-U_{o}$ and *k* stands for the Boltzmann constant. The calculated value of *p* was compared with a uniformly distributed random number r∈(0,1). If r<p, the new configuration was accepted; in the opposite case, the selected atom or molecule was moved back to the initial (old) position on the lattice and the next MC step was performed. In the simulation, typically, $N\times 10^{5}$ steps were made at a given *T*, the last 10% of which were used for the calculation of average values. Moreover, to minimize the risk of trapping the modeled systems in metastable states, gradual cooling of the adsorbed overlayer was implemented. During this procedure, the temperature was linearly decreased from 0.51 to 0.01 using 500 steps of equal length.

Surface coverage *θ* was defined as the average number of adsorbed segments per lattice site, that is, $(4N_{l}+N_{m})/L^{2}$. Quantitative descriptors of the simulated systems presented here, including coordination functions, cluster size distributions, and order parameters, are averages over ten independent replicas.

Energies and temperatures of our model were expressed in the units of ε and $\left|\varepsilon \right|/k$, respectively. Precisely, the values of system energy and temperature are dimensionless and have no physical units. Instead, they are real numbers that specify how many times the elementary portion of energy, ε, and the temperature, |ε|/*k*, fit into the simulated values of energy and temperature, *T*, respectively. Note that ε can be given in any energy units (J, cal, eV), so that the units of temperature |ε|/*k* are automatically adjusted. To maintain the broad applicability of the model, we decided to use only numerical values for the parameters and leave the choice of units to the reader, depending on the specific system. For that reason, our model is dependent on just one working parameter, ε, which describes the single metal–linker interaction and whose numerical value was assumed to be −1. Accordingly, the reduced Boltzmann constant, *k*, was set to 1 to make temperature, *T*, dimensionless.

In summary, the input parameters in our model are *L*, *ε*, *N*, and *T*, with the first two being fixed in all of the calculations, that is L=200 and ε=−1. The targeted parameters include the orientational parameters *δ_a_* and *δ_c_*, the fractions of homochiral (*S*-*S* and *R*-*R*) and heterochiral (*R*-*S*) metal-mediated links, and the statistics of aggregate size. The parameters *δ_a_* and *δ_c_* were determined by counting the number of molecules with their arms (a) and cores (c) oriented in the three primary directions of the triangular lattice. Figure S5 in the Supporting Information section illustrates the definitions of the core- and arm-based orientation directions. The determined numbers of molecules with distinct orientations were inserted into Equation (S1) in that section. The fraction of homo- and heterochiral links was calculated by accounting for metal atoms coordinated with two enantiomers of the same type for homochiral links and of opposite types for heterochiral links. These numbers were divided by the total number of metal atoms in the system (*N_m_*) to yield the corresponding fractions. Examples of bimolecular configuration of the aforementioned types are presented in Figure S4 in the Supporting Information Section. The statistics of aggregate size were determined based on the number of clusters consisting of a specific number of molecules (ranging from 1 to *N_l_*), which were calculated using a suitably modified Hoshen–Kopelman algorithm for cluster identification [39].

The algorithm employed in our calculations implemented the conventional Metropolis sampling in the canonical ensemble [39]. The lattice of adsorption sites was represented as a square *L* × *L* table, with each cell being characterized by the occupation variable *s*, which could take on values of 0 (representing an unoccupied site), 1 (indicating occupancy by a molecular segment), or 2 (denoting occupancy by a metal atom). The nearest neighbors of a specific cell in the table were redefined to include six cell sites, corresponding to the topology of a triangular lattice. The simulation began with a collection of randomly distributed molecules (collections of cells with s=1) and metal atoms (single cells with s=2) in quantities *N_l_* and *N_m_*, respectively. To adsorb a molecule, a cluster of four sites matching the shape of a given chrysene tecton was selected, as illustrated in Figure 7. The distance between any pair of neighboring segments of the molecule was equal to $a\sqrt{3}$, corresponding to the next-nearest-neighbor distance on a triangular lattice. The occupation variables *s* of the cells corresponding to these sites were all set to 1. In the case of a metal atom, a single cell was chosen, and the associated value of s was updated to 2. To determine if there exists an effective interaction between an adsorbed molecule and a metal atom, the active molecular segments were probed. To that end, the next-nearest-neighbor site of a selected active segment was searched out in the direction determined by the specific position of the halogen substituent. If the occupation variable *s* of this site was equal to 2, then a further search in the same direction was made at the next step of $a\sqrt{3}$, aiming to determine if a second molecule could be linked with the metal atom. If for that site s was zero, then the first molecule interacted with the metal atom with energy ε, providing single metal coordination. If s was equal to one, the match between the interaction directions (halogen substituents) of the first and second molecule was checked. If the interaction directions were collinear (→←), the second molecule contributed with an additional input of ε, resulting in double coordination of the metal atom with energy 2ε. If the interaction directions were not collinear, the energy of this configuration was reduced and equal to ε. For all other situations in which the occupation variables associated with neighboring segments were not equal to 1 (linker) and 2 (metal), the interaction energy was zero. An analogous scheme was used for a metal atom if this component was selected in the equilibration procedure. In this case, a single lattice site was considered, so the occupation variable for an adsorbed metal atom was set to 2.

To account for the interactions with neighboring molecules, six next-nearest-neighbor sites of the metal atom were checked (at a distance of $a\sqrt{3}$ each). If any of these sites were occupied by an active molecular segment, the directionality of the interaction provided by this molecular segment (halogen position) was inspected. If the interaction direction assigned to the segment pointed towards the metal atom, a single link was formed with energy ε. Then, the second site of the six surrounding the metal atoms was examined, and if this site was occupied by an active molecular segment, the same procedure was used to determine the corresponding interaction direction. If this interaction direction was collinear with that of the first molecule, the second link was formed, contributing to ε. For the 120° alignment of interaction directions, the energy was equal to ε. No more than two linkers were allowed to link with a single metal atom, and this condition was always checked when updating the coordination of a metal atom. The energy of a selected molecule or a metal atom was determined using the method described above, and it served as the input $U_{o}$ (old configuration) or $U_{n}$ (new configuration) for the previously mentioned Metropolis acceptance criterion.

All computer programs utilized in this study were developed from scratch using the FORTRAN programming language. The calculations were carried out on a computer cluster running the Linux operating system. Executable files were generated using the gfortran GNU compiler.

## 4. Summary and Conclusions

The results of this study demonstrated the effectiveness of the simplified coarse-grained MC model in predicting possible structures of metal-organic intermediates created in the on-surface Ullman coupling of brominated chrysene isomers. In particular, the model was revealed to be able to correctly reproduce the self-assembly of 6,12-dibromochrysene (**ch6_12**) adsorbed on Cu(111). Based on this outcome, our theoretical investigations were extended to three additional doubly halogenated chrysene tectons. The simulations showed that the chain formation observed for **ch6_12** can be redirected towards cyclic oligomers by a suitable modification of the halogenation pattern. Specifically, for the additional isomers, the unique distribution of halogen atoms resulted in the formation of small triangular aggregates (**ch36**) and larger hexameric units having regular ring (**ch26**) and star shapes (**ch6_11**).

Important information gained from the simulations that would not be easily accessible from the experiment was the influence of the enantiomeric composition of the adsorbed phase on the main structural properties of the resulting precursors. The simulations performed in the adsorbed overlayers comprising one surface enantiomer of a given **ch** derivative revealed significant morphological changes induced by the introduction of the mirror-image component (*R*). In general, in the adsorbed racemic mixtures of **ch** (which should occur naturally in most experimental situations due to unbiased adsorption of the enantiomers), the presence of *S* induced considerable structural disordering and deterioration of the quality of the obtained connections. This was clearly visible, for example, in the case of *rac*-**ch6_11**, where the enantiomers formed winding chains with random *S*/*R* sequences instead of the uniform star-shaped hexamers created in the corresponding enantiopure system. The increased structural heterogeneity was also observed for the racemate of **ch26** comprising six-membered loop oligomers of a few distinct shapes, imperceptible when one enantiomer was at play. Similarly, for the trimer-forming molecule **ch36**, the enantiomers, apart from recreating the homochiral oligomers, arranged into additional triangular aggregates with reduced symmetry. In the case of the experimental example related to dibromochrysene molecules, the simulations showed that the hypothetical enantiopure self-assembly would produce chain polymers with less deformed shape as compared to the natural racemic conditions. These findings were in agreement with the experimentally observed homochiral *S* and *R* chains formed on Cu(111) due to the incommensurate arrangement of adatoms [28].

Regarding the effect of model parameters on the obtained metal–organic connections, it was found that the structural transitions resulting in the effective formation of the corresponding aggregates occur for temperatures typically varying from 0.15 to 0.25. These onset values were influenced by the surface coverage, and for lower surface coverage (θ=0.0125, corresponding to 100 adsorbed molecules), they were shifted towards the lower limit. For denser adsorbed systems, the chances of finding interacting species were increased, resulting in a higher onset temperature. This is clearly visible for the trimer-forming tecton **ch36**. Moreover, a similar effect was observed for the chain-forming isomer **ch6_12**, where the molecules were strongly confined by already formed long chains, forcing them to adopt identical structures. The surface coverage also had a significant influence on the order parameters *δ_a_* and *δ_c_*, which characterize the degree of uniform alignment of adsorbed molecules. High values of these parameters, close to 1, were recorded for 700 molecules of **ch6_12** (θ=0.0875), which exclusively formed chains in both enantiopure and racemic overlayers. On the other hand, for isomers capable of creating cyclic structures such as trimers and hexamers, the parameters δ_a_ and δ_c_ were usually no greater than 0.05, indicating an equal contribution of the allowed molecular orientations to these structures.

The findings presented in this work showed that different isomers of dihalogenated chrysene can potentially be used for synthesizing macrocyclic compounds of various shapes and sizes. The trimeric cyclic units predicted for **ch36** and the hexagonal hexamers obtained with **ch6_11** demonstrated the weak influence of the surface coverage on the morphology of these metal-organic structures. In practice, this means a wide range of surface coverage changes enable the synthesis of the mentioned macrocycles. On the contrary, at higher adsorbate density, the formation of hexagonal oligomers of **ch26** was suppressed in favor of closely packed chains. The trimers of **ch36** had an identical pore shape, regardless of whether they were formed in the enantiopure or racemic overlayer, indicating that these triangular connections do not require enantiopurity of the adsorbed phase (which is much harder to guarantee in the experiment). However, to obtain uniform hexamers of **ch36** and **ch6_11**, enantiopurity is required, as the presence of opposite surface enantiomers of these tectons drastically deteriorates the quality of the formed connections. In practice, the enantiopurity of **ch36** and **ch6_11** can be achieved by additional functionalization of these molecules, which would force them to adopt surface conformations of one kind. The structures obtained in this study can have a potential impact on the design of macrocycles with pore rims decorated with diverse functional groups. Based on the architecture of the cyclic units simulated here, it can be deduced, for example, where to introduce extra groups in a given chrysene isomer to place them either inside or outside the pore. In this context, the proposed methodology can be relevant to the on-surface synthesis of chiral macrocyclic molecules with differently modified (activated/deactivated) zones.

One of the potential application areas of this approach is designing low-dimensional polymers with special properties, such as electrical, optical, and catalytic properties. The materials of this type include (super)conducting polymers, layered materials with unique optical/reflecting properties (chiral overlayers), and porous networks (also chiral) for selective adsorption and transformation of guest species in confined spaces (heterogeneous catalysis).

The approach based on metal-mediated directional interactions can be utilized for modeling the self-assembly and subsequent reaction of larger molecules equipped with discrete interaction centers. This also applies to molecular bricks used for the synthesis of bioactive compounds (e.g., amino acids) [40,41]. For example, copper atoms on an adsorbing surface can compel interacting molecules to form organometallic connections with specific geometries. As a result, the synthesis can be directed towards producing a molecule with the desired structure and (bio)activity, which may not be achievable using conventional bulk reactions. The coarse-grained Monte Carlo model proposed here can help predict the molecular connections formed under two-dimensional confinement.

For future applications, the results of our theoretical studies can be helpful in the preliminary selection of chrysene-based building blocks for the directed on-surface synthesis of covalent polymeric structures with predefined properties. In particular, the metal-organic assemblies reported herein can facilitate the choice of initial guess of the halogenation pattern to obtain persistent molecular architectures with controlled topology, structural heterogeneity, and chirality. These predictions can thus substantially reduce the number of trial experiments required to select the optimal tecton, including test syntheses and STM inspections of the corresponding superstructures. Moreover, the proposed methodology can be easily extended to other molecular tectons having diverse shapes/sizes and number and spatial distribution of halogen substituents.

## Data Availability

Data used in this paper is available to view by contacting authors.

## Supplementary Materials

The following supporting information can be downloaded at: https://www.mdpi.com/article/10.3390/molecules29071553/s1, Figure S1: Simulated enantiopure and racemic overlayers comprising 300*S* and 500*S* and 150*R* + 150*S* and 250*R* + 250*S* molecules of **ch6_12** mixed with 300 and 500 metal atoms; Figure S2: Relative abundance of trimolecular aggregates of **ch36** having different enantiomeric compositions; Figure S3: Simulated enantiopure and racemic overlayers comprising 100*S* and 50*R* + 50*S* molecules of **ch26** mixed with 100 metal atoms; Figure S4: Energies of the mono- and bimolecular metal-mediated links assumed in the simulations; Figure S5: Schematic illustration of the core and arm reference directions used in the definition of the orientational order parameters and *δ_a_* and *δ_c_*.

## Abbreviations

∆*U*	energy difference (∆*U* = *U_N_* − *U_O_*)
2D	two-dimensional
*a*	lattice constant (equals 1)
*A*	system area
Ag	silver
Au	gold
**ch**	chrysene
**ch26**	2,6-di-X-chrysene
**ch36**	3,6-di-X-chrysene
**ch6_11**	6,11-di-X-chrysene
**ch6_12**	6,12-di-X-chrysene
Cu	copper
DBCh	6,12-dibromochrysene
DFT	density functional theory
IUPAC	International Union of Pure and Applied Chemistry
*k*	reduced Boltzmann constant (equals 1)
*L*	system side length
MC	Monte Carlo method
MD	molecular dynamics
*N*	total number of species
*N_l_*	number of molecules
*N_m_*	number of metal atoms
*p*	acceptance probability ($p=\min\;[1,\exp\left(-\frac{∆U}{kT}\right)])$
PAH	polyaromatic hydrocarbon
*r*	comparison number (*r* = random(0,1))
*R*, *S*	conformations of adsorbed prochiral molecules
*rac*	racemic mixture
STM	scanning tunnelling microscopy
*T*	temperature
*U_N_*	potential energy of a new configuration
*U_O_*	potential energy of an old configuration
*δ*	order parameter
*δ_a_*	arm-based order parameter
*δ_c_*	core-based order parameter
*ε*	energy of single metal-linker interaction (equals −1)
*θ*	surface coverage ($\theta =(4N_{l}+N_{m})/L^{2}$)
