# Supplementary material for: Predicting Organometallic Intermediates in the Surface-Assisted Ullmann Coupling of Chrysene Isomers
Source: Molecules. 2024 Mar 30;29(7):1553. doi: 10.3390/molecules29071553 (PMC11013314; doi:10.3390/molecules29071553)
Supplement: Supplementary file 1 [file molecules-29-01553-s001.zip › molecules-2901885-supplementary.pdf]

Supporting Information

for

Predicting Organometallic Intermediates in the Surface-Assisted Ullmann Coupling of Chrysene Isomers

Jakub Lisiecki and Paweł Szabelski*

Department of Theoretical Chemistry, Institute of Chemical Sciences, Faculty of Chemistry, Maria Curie-Skłodowska University in Lublin, Pl. M.C. Skłodowskiej 3, 20-031 Lublin, Poland

Orientational order parameter

The orientational order parameter was calculated as the ensemble average, using the following equation:

$$\delta = \frac{|N_{l1} - N_{l2}| + |N_{l2} - N_{l3}| + |N_{l1} - N_{l3}|}{2 * N_l} \qquad \textbf{Eq. S1}$$

where N_{l1}, N_{l2}, N_{l3} are the average numbers of linkers oriented along the 3 main directions of a triangular lattice, and N_l is the total number of linkers in the system. As every linker in the pair is counted twice, the result is divided by 2. For δ_a (arm-based) the orientation of a linker was determined by the relative position of the first two segments, for δ_c (core-based) – the orientation was determined by the position of two middle segments (as shown in Fig. S1).

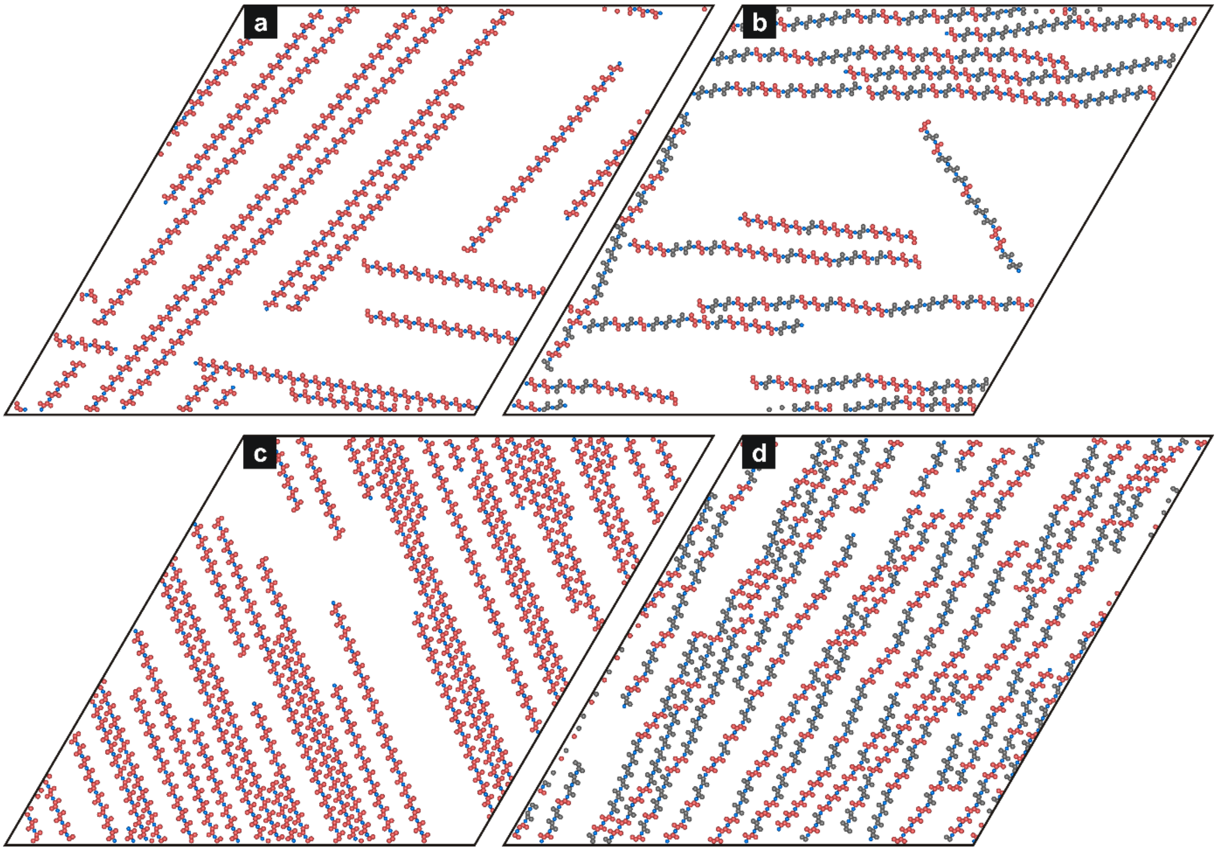

Figure S1. Simulated enantiopure (a, c) and racemic (b, d) overlayers comprising 300*S* (a) and 500*S* (c) and 150*R* + 150*S* (b) and 250*R* + 250*S* (d) molecules of **ch6_12** mixed with 300 and 500 metal atoms, respectively; $T = 0.01$.

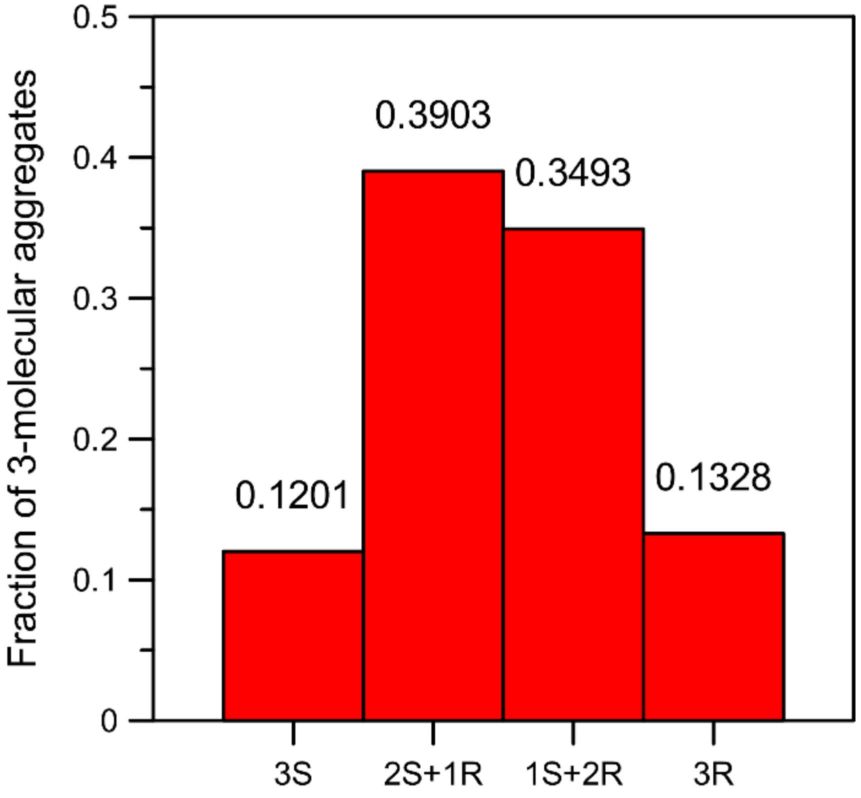

Figure S2. Relative abundance of trimolecular aggregates of **ch36** having different enantiomeric compositions (N_l = 400, T = 0.01).

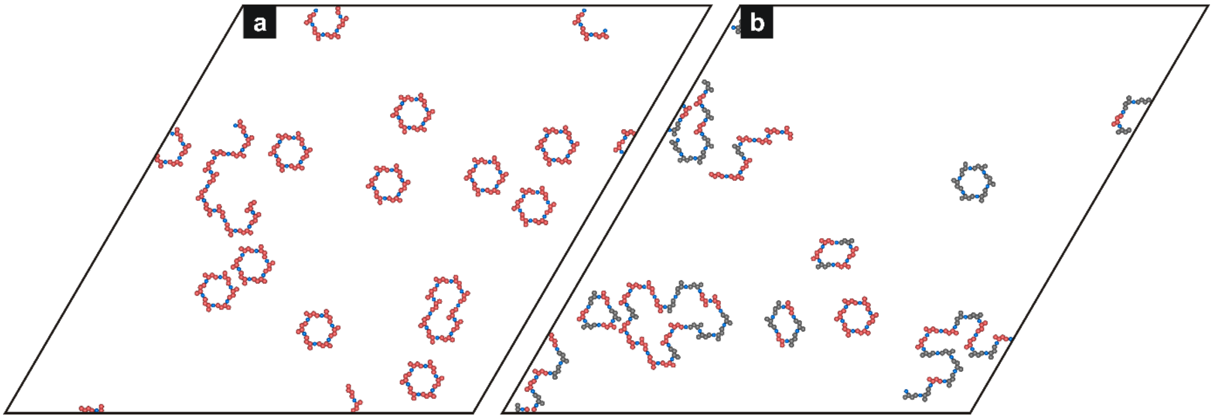

Figure S3. Simulated enantiopure (a) and racemic (b) overlayers comprising 100*S* (a) and 50*R* + 50*S* (b) molecules of **ch26** mixed with 100 metal atoms; T = 0.01.

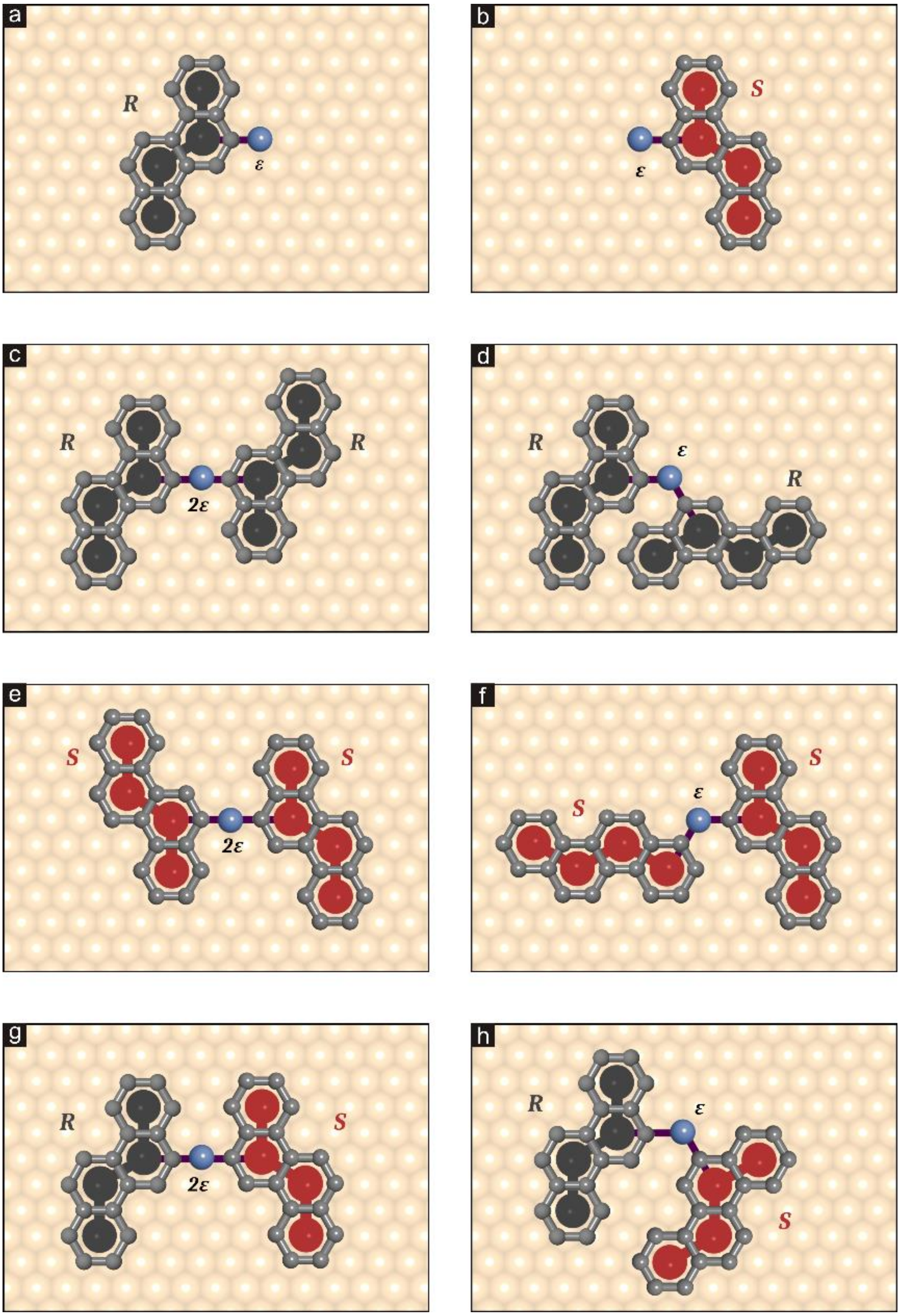

Figure S4. Energies of the mono- and bimolecular metal-mediated links assumed in the simulations. Metal atoms are depicted in blue while the enantiomers *R* and *S* are colored in grey and red, respectively. Single molecular connections (a, b) contribute with energy ε, while linear (c, e, g) and 120° (d, f, h) bimolecular nodes contribute with, 2ε and ε, respectively.

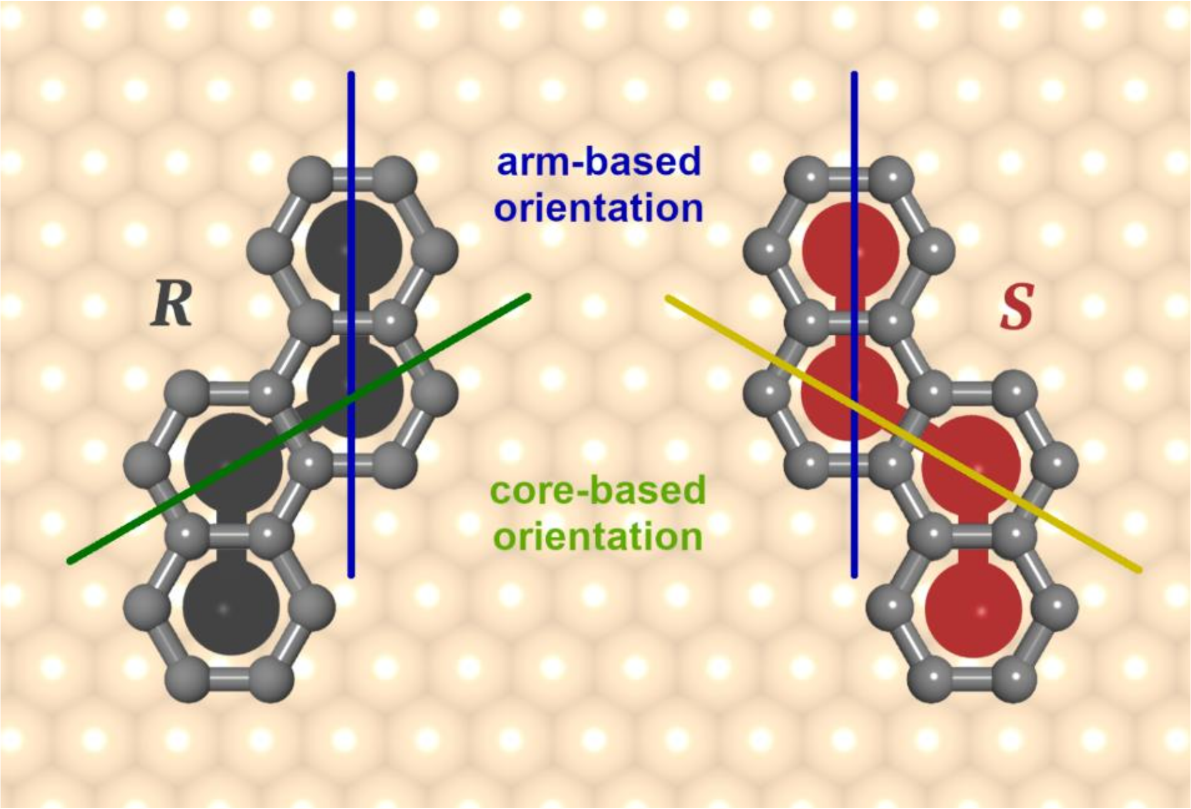

Figure S5. Schematic illustration of the core (shades of green) and arm (blue) reference directions used in the definition of the orientational order parameters and δ_c and δ_a , respectively.
